# HCK and ABAA: A Newly Designed Pipeline to Improve Fungi Metabarcoding Analysis

**DOI:** 10.3389/fmicb.2021.640693

**Published:** 2021-05-05

**Authors:** Kodjovi D. Mlaga, Alban Mathieu, Charles Joly Beauparlant, Alban Ott, Ahmad Khodr, Olivier Perin, Arnaud Droit

**Affiliations:** ^1^Department of Molecular Medicine, Laval University, Quebec, QC, Canada; ^2^Centre de Recherche du CHU de Québec, Quebec, QC, Canada; ^3^Research and Innovation, L’Oreal, Paris, France

**Keywords:** ABAA, benchmarking, F-score, fungi, HCK, hierarchical clustering, ITS amplicons

## Abstract

**Introduction:**

The fungi ITS sequence length dissimilarity, non-specific amplicons, including chimaera formed during Polymerase Chain Reaction (PCR), added to sequencing errors, create bias during similarity clustering and abundance estimation in the downstream analysis. To overcome these challenges, we present a novel approach, Hierarchical Clustering with Kraken (HCK), to classify ITS1 amplicons and Abundance-Base Alternative Approach (ABAA) pipeline to detect and filter non-specific amplicons in fungi metabarcoding sequencing datasets.

**Materials and Methods:**

We compared the performances of both pipelines against QIIME, KRAKEN, and DADA2 using publicly available fungi ITS mock community datasets and using BLASTn as a reference. We calculated the Precision, Recall, F-score using the True-Positive, False-positive, and False-negative estimation. Alpha diversity (Chao1 and Shannon metrics) was also used to evaluate the diversity estimation of our method.

**Results:**

The analysis shows that ABAA reduced the number of false-positive with all metabarcoding methods tested, and HCK increases precision and recall. HCK, coupled with ABAA, improves the F-score and bring alpha diversity metric value close to that of the BLASTn alpha diversity values when compared to QIIME, KRAKEN, and DADA2.

**Conclusion:**

The developed HCK-ABAA approach allows better identification of the fungi community structures while avoiding use of a reference database for non-specific amplicons filtration. It results in a more robust and stable methodology over time. The software can be downloaded on the following link: https://bitbucket.org/GottySG36/hck/src/master/.

## Introduction

The mycobiome concept was first introduced in 2010 to designate the fungal community of the human oral cavity (Tang et al., 2015) before being extended to other micro-environments. Three genomic markers are widely used to identify fungal species in a microbial environment: 18S ribosomal gene (Wu et al., 2015), 28S ribosomal gene (Ninet et al., 2003), and the Internal Transcribed Spacers (ITS) (Martin and Rygiewicz, 2005; Bellemain et al., 2010). The most commonly used is the ITS amplicon (Fujita et al., 2001) which targets two loci: ITS1, located between the 18S and 5.8S genes, and ITS2, between 5.8S and 28S (Bellemain et al., 2010). ITS1 has been demonstrated to yield the best performance (Bazzicalupo et al., 2013; Wang et al., 2015). Several packages have been developed to automate the process, and most of them are OTU (Operational Taxonomic Unit) sequence similarity-based pipeline (Schloss et al., 2009; Gweon et al., 2015; Rognes et al., 2016; Mysara et al., 2017; Bolyen et al., 2018). To date, the research communities are gradually moving to the new concept of ASVs (Amplicons sequence Variants) or Exact Sequences Variants (ESVs) (Callahan et al., 2017). With these pipelines, the taxonomy delineates based on the single nucleotides’ variant of amplicons, assuming that amplicons sequences have a similar length which is not the case with fungi ITS sequences. To date, several pipelines have been developed to classify fungal species using ITS sequencing. These include Plutof (Abarenkov et al., 2010b), Clotu (Kumar et al., 2011), PIPITS (Gweon et al., 2015), CloVR-ITS (White et al., 2013), and BioMaS (Fosso et al., 2015) specially designed to analyse fungi ITS datasets, Kraken (Wood and Salzberg, 2014), Mothur (Schloss et al., 2009) Qiime (Caporaso et al., 2010; Bolyen et al., 2018), Vsearch (Rognes et al., 2016), and DADA2 (Callahan et al., 2016) among many others, to examine both bacterial 16S rRNA and fungal ITS amplicons.

The size of fungal ITS sequences is highly variable, and species can differ widely by the number of loci (Tang et al., 2015; Khodadadi et al., 2017). The sequence length dissimilarity creates bias during clustering and affects OTUs abundance estimation. Moreover, besides biologically valid amplicons, PCR generates many non-specific fragments resulting from elongation interruption or two or more incomplete amplicons joining (chimaeras) (Lahr and Katz, 2009; Edgar, 2016; Bjørnsgaard Aas et al., 2017). These non-specific amplicons are hybrid products between multiple parent sequences that can be falsely interpreted as existing or novel species, thus significantly affect the diversities, including the alpha and beta diversity metrics (Zajec et al., 2012). Hence, non-specific amplicons formed during amplification with two incomplete segments (*bimeras*) are generally at a lower proportion. However, chimaeras with more than two fragments (*multimers*) may form at comparable rates and account for a significant fraction in an amplified sample (Lahr and Katz, 2009). The most commonly used pipeline to detect chimaeras is UCHIME, composed of reference-based and *de novo* approaches (Edgar, 2016). The reference-based approach detects non-specific amplicons in a dataset by making a model from a concatenated pair of sub-sequences in a reference database. Chimaeras are detected if the query alignment sequence score of the model exceeds a threshold. UCHIME depends on a reference database, and ITS sequence size variation can be a significant source of false-positive detection, throwing away biologically valid sequences. DADA2 implements *isBimeraDenovo()* function that identifies exact bimeras or multimeras sequences. Child sequences that differ by a single mismatch from the chimeric model are flagged if the left parent and right parent are at least four nucleotides away from the child sequence (Callahan et al., 2016). The challenge is that databases are rarely updated, and the similarity search can be time-consuming, especially when databases are large. Computational resources are one of the critical limitations. Maintaining specific databases up to date is a real challenge, and a broad range of databases suffer from contamination and unannotated sequences. The available databases, such as UNITE, which is commonly used, presents 26% of entries that cannot be consistently assigned to a taxonomic family (Nilsson et al., 2008; Kõljalg et al., 2013). These tools are mainly developed for 16S/18S markers but widely applied to fungal ITS amplicons. Besides, these tools have been optimised using simulated datasets and not real datasets (Bjørnsgaard Aas et al., 2017).

To overcome the above limitations, we present a novel classification approach for ITS amplicon’s taxonomy assignment. This approach consists of two steps: The amplicons Abundance-Base Alternative Approach (ABAA), a *de novo* method to filter non-specific amplicons from sequence datasets and a Hierarchical Clustering with Kraken (HCK) to classify ITS amplicons. We built HCK on a hierarchical clustering approach with multiple-step iterating runs. Each cluster’s representative sequences are taxonomically assigned using *Kraken* with the exact alignment of k-mers using fungal ITS loci sequence database (ITSdb). In this study, we use comparative analysis approach to assess the performance of ABAA and HCK. We calculated the Precision, the Recall, and the F-score using the True-Positive, False-positive, and False-negative estimation. Alpha diversity (Chao1 and Shannon) was also used to evaluate the methods’ diversity estimation. Chao1 is based on the concept that rare species allow inferring the number of missing species. As the Chao1 richness estimator gives more weight to the low abundance species while the Shannon index measures the richness and the evenness (Kim et al., 2017), making the Chao1 metric more sensitive to abundance estimation than Shannon’s. Henceforth, to simplify the manuscript, chimaeras and non-specific amplicons will interchangeably be used to designate all non-specific amplicons, including chimaeras, incomplete amplicons and sequencing errors.

## Materials and Methods

The methodology in this study is organised in two parts. In the first part, we will describe ABAA and HCK workflow using publicly available ITS mock community datasets. We will then, in a second part, compare the performance of HCK-ABAA to that of QIIME, DADA2 and KRAKEN using BLASTn search abundance estimation as a reference.

### Fungi ITS Mock Communities’ Datasets

We downloaded Biological mock community datasets of three different projects from the SRA NCBI database. The three projects were conducted using the Illumina Miseq sequencing technology. The first project, available under accession number *PRJNA516455* (McTaggart et al., 2019), contains six different samples (*SRR8473974, SRR8473977, SRR8473978, SRR8473979, SRR8473980, SRR8473984*), which were prepared from subsets of 53 species of fungi with an emphasis on human lung pathogens. The second project, available under accession number *SRP132544* (Hoggard et al., 2018), contains three samples (*SRR6702280, SRR6702281, SRR6702283)*, including specific fungal species from different human body location or organs (lung, oral cavity, gastrointestinal tract, and skin). The third project, available under accession number *PRJNA382746*, contains two samples (*SRR5439721, SRR5439722)* that include 16 species of fungi. Overall, the mock communities contain 36 fungi genera which are: *Alternaria, Apophysomyces, Aspergillus, Blastomyces, Candida, Cladosporium, Clavispora, Coccidioides, Cryptococcus, Cunninghamella, Exophiala, Fusarium, Histoplasma, Lichtheimia, Malassezia, Meyerozyma, Mucor, Paecilomyces, Penicillium, Phanerochaete, Pichia, Purpureocillium, Rasamsonia, Rhizopus, Saccharomyces, Sarocladium, Scedosporium, Schizosaccharomyces, Sporidiobolus, Sporothrix, Talaromyces, Trichoderma, Trichosporon, Wickerhamomyces, Sclerotina, Rhyzomucor, Trichophyton* detailed in Table 1.

**TABLE 1:** Absolute count of reads affiliated of each genus among the different datasets of the mock community (determined by BLASTn against NT database).

Taxa	SRR 5439721	SRR 5439722	SRR 6702280	SRR 6702281	SRR 6702283	SRR 8473974	SRR 8473977	SRR 8473978	SRR 8473979	SRR 8473980	SRR 8473984
*Alternaria*	0	0	3,941	3,334	5,462	39,009	0	0	0	0	0
*Apophysomyces*	0	0	0	0	0	0	0	0	0	0	191
*Aspergillus*	0	1,381	924	1,029	1,120	315,986	5,991	5,096	5,657	3,613	2,362
*Blastomyces*	0	0	0	0	0	0	379	230	256	183	0
*Candida*	150,704	108,934	33,556	28,123	39,320	125,949	0	0	0	0	0
*Cladosporium*	0	0	51	66	76	22,476	0	0	0	0	0
*Clavispora*	0	0	0	0	0	1,645	0	0	0	0	0
*Coccidioides*	0	0	0	0	0	0	502	649	698	515	0
*Cryptococcus*	1,087	33,983	12	9	16	16	555	568	825	665	131
*Cunninghamella*	0	0	0	0	0	0	0	0	0	0	13
*Exophiala*	0	0	3,340	2,791	4,140	0	299	271	319	221	2
*Fusarium*	0	0	52	54	76	39,505	0	0	0	0	138
*Histoplasma*	0	0	0	0	0	0	152	112	138	87	0
*Lichtheimia*	0	0	0	0	0	0	0	0	0	0	45
*Malassezia*	0	0	50	67	67	0	0	0	0	0	0
*Meyerozyma*	0	0	0	0	0	15,220	0	0	0	0	0
*Mucor*	0	0	0	0	0	11,935	109	96	109	80	438
*Paecilomyces*	0	0	0	0	0	0	0	0	0	0	80
*Penicillium*	0	0	32,037	29,551	39,368	27,888	1,645	1,461	1,593	854	0
*Phanerochaete*	32,650	20,763	0	0	0	0	0	0	0	0	0
*Pichia*	22,965	69,159	0	0	0	95,622	0	0	0	0	0
*Purpureocillium*	0	0	0	0	0	0	120	120	128	96	0
*Rasamsonia*	0	0	2	0	0	292	0	0	0	0	128
*Rhizopus*	0	0	0	0	0	57	2,283	2,354	3,447	2,584	109
*Saccharomyces*	57,107	46,582	1,737	1,863	1,942	12,654	0	0	0	0	0
*Sarocladium*	0	0	0	0	0	0	257	269	336	221	0
*Scedosporium*	0	0	0	0	0	3	62	75	86	54	36
*Schizosaccharomyces*	36,413	23,880	0	2	0	7	0	0	0	0	0
*Sporidiobolus*	0	0	0	0	0	17,476	0	0	0	0	0
*Sporothrix*	0	0	0	0	0	2	0	0	0	0	1
*Talaromyces*	0	0	0	0	0	1,336	435	382	403	160	6
*Trichoderma*	29,027	18,431	0	0	0	0	0	0	0	0	0
*Trichosporon*	0	0	122	77	185	88,790	2,305	2,080	2,221	1,642	0
*Wickerhamomyces*	2,208	1,235	0	0	0	10	0	0	0	0	0
*Sclerotina*	25,545	12,058	0	0	0	0	0	0	0	0	0
*Rhyzomucor*	0	0	0	0	0	0	0	0	0	0	0
*Trichophyton*	0	0	7,142	6,084	9,087	0	0	0	0	0	0

### Fungi ITS Analysis Workflow With HCK-ABAA

#### Data Pre-processing and Quality Check

The sequence reads are trimmed with paired-end mode using *Trimmomatic* (Bolger et al., 2014) to remove residual adapters. The default parameters are used, including “phred33” to encode the quality part of the Fastq file to base 33, the low-quality bases from the sequence beginning and the end is set to 3 bases, respectively. The sliding window size was set to 4 with a minimum length of 50 bases. The paired reads generated from the trimming are then joined into contigs to produce the final fasta file using *Pandaseq* (Masella et al., 2012) with default parameters. Sequences with ambiguous bases are removed.

#### Non-specific Amplicons Filtering: ABAA

We empirically consider that amplicons with length-frequency below the standard deviation overall distribution to originate from non-specific amplification. Technically, after determining the amplicons’ length distribution and their frequency within each sample, an amplicon is considered to be non-specific if its length-frequency is below a certain threshold. This threshold corresponds to the standard deviation of the frequency of the amplicon lengths. ABAA filtering corresponds to step 1 of the whole pipeline.

#### Hierarchical Clustering With Kraken Assignment (HCK)

##### Amplicons Hierarchical Clustering

**FIGURE 1:**
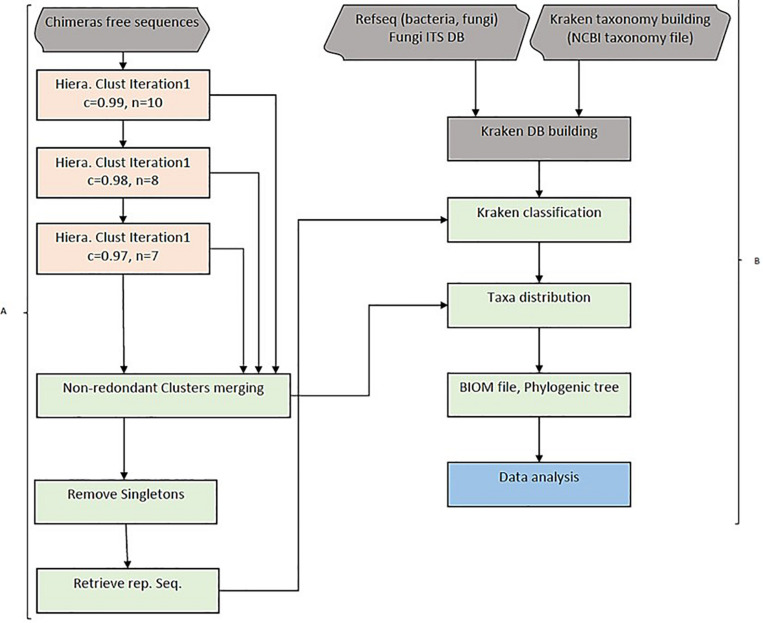
HCK workflow diagram. **(A)** Hierarchical clustering with three iterations. Chimaeras free sequences are results of pipeline step 1, including raw reads trimming, merging (forward and reverse reads) and ABAA filtering. Sequences are combined into one single fasta file and clustered using a hierarchical clustering approach in step 2. All clusters are then merged into one single non-redundant clusters and got rid of singletons sequences. HCK retrieves representative sequences from each cluster for amplicons’ classification, the second part of the pipeline (step 3). **(B)** Classification uses Lowest Common Ancestor (LCA) taxonomical assignment implemented in Kraken to classify representative sequences and taxonomy reported to each cluster, and a final BIOM file can be generated for downstream analysis (Steps 4 and 5).

Amplicon hierarchical clustering corresponds to step 2 of the whole pipeline. HCK clusters amplicons sequences using multiple-step iterated runs of sequence alignments with a neighbour-joining algorithm implemented in CD-HIT version 4.5.4 (Fu et al., 2012). A segment sliding window in this context or “word” is defined as the consecutive position of a certain number of nucleotides in a sequence fragment. We implemented three iterative runs in the clustering and set the sequence identities (c) to 0.99, 0.98, and 0.97, as well as the “*word”* size (n) to 10, 8, and 7 bps, respectively. It is possible to control the sequence length difference cut-off(s), the alignment coverage of the more extended sequence (aL), and the alignment coverage for the shorter sequence (aS). The most crucial parameter is the length difference cut-off(s) depending on the overall distribution of the amplicon’s size. It can be empirically estimated by dividing the average size by the size of the most extended amplicon. This value was set to 90% in the study. The iterated clusters generated are then merged into one single, no redundant cluster file and sorted by size to remove singleton amplicons. An intermediary step 3 is essential to retrieve representative sequences from each cluster and be classified using Kraken (Figure 1A).

##### ITS Loci RefSeq

We downloaded the fungal Internal Transcribed Spacer RNA (ITS) RefSeq Targeted Loci (ITSdb) containing 11,252 entries. We retrieved the corresponding taxonomy profile from the NCBI taxonomy database^1^ and created a Qiime-compatible taxonomy file. Both files (fasta and taxonomy file) were sorted and cleaned to have similar entries, using the following utilities^2^. ITSdb was used to generate a kraken database following the procedure available at this web address: http://ccb.jhu.edu/software/kraken/MANUAL.html.

##### Taxonomical Classification

Each cluster’s representative sequences are classified using the Lowest Common Ancestor (LCA) algorithm with Kraken version 1 (Wood and Salzberg, 2014). The taxonomy assignment is then extended to other amplicons of the respective clusters for a complete classification. This step corresponds to step 4 of the HCK workflow. The command uses the sample metadata information to generate a BIOM file. The final stage, step 5, uses the BIOM file to estimate the diversity abundance and further metric calculation analysis (Figure 1B).

### Benchmark Analysis and Performances Evaluation

#### BLASTn (Reference)

We determined the actual reference diversity and abundance with BLASTn sequence similarity search against the NCBI NT database. Consensus classification was determined for coverage ≥98%, identity ≥97%, and *e*-value ≤ 0.00001 with a maximum of 100 hits retained per entry. The BLASTn output was then filtered for the best hits successively by the *e*-value, coverage percentage, and identity percentage. The final consensual taxonomy classification for each amplicon is kept based on a minimum number of 80 identical taxid out of 100 for each query (80% of the total hits) to generate an abundance table following a procedure described by other authors (Blaalid et al., 2013; McTaggart et al., 2019).

#### Comparative Analysis

**FIGURE 2:**
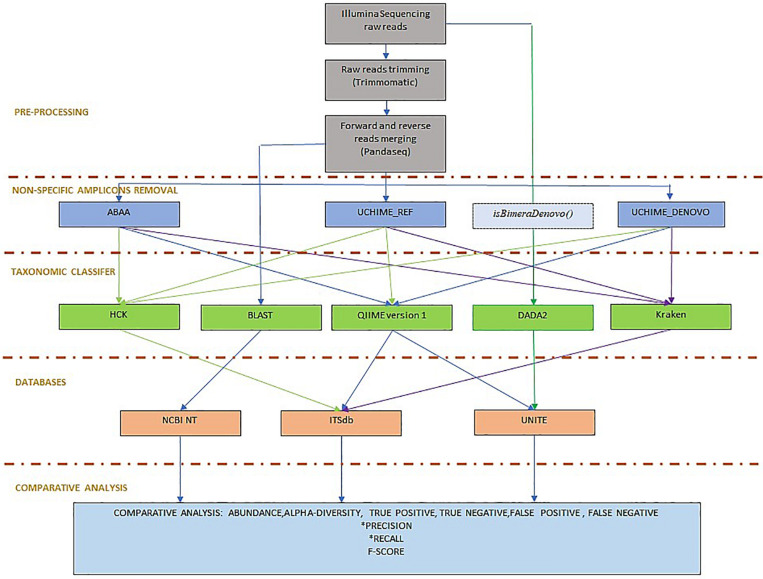
Benchmarking workflow: the workflow is organised in pre-processing, including Illumina sequencing reads trimming and forward and reverse reads merging. To determine the best non-specific amplicons filtering method, ABAA was tested with UCHIME (UCHIME_Ref, UCHIME_DENOVO, *and isBimeraDenovo()* implemented in DADA2. The classifier tested also includes HCK with ITSdb, the newly designed pipeline, QIIME with ITSdb and UNITE, Kraken with ITSdb, DADA2 with UNITE and compared to BLASTn search using NCBI NT database, as reference. The pipelines performances are evaluated using precision, recall and F-score.

To evaluate the efficacy of the newly developed tools, we compared the absolute count diversity of HCK to Qiime v1.9 (Caporaso et al., 2010), Kraken (Wood and Salzberg, 2014), and DADA2 (version 1.8) (Callahan et al., 2016) with and without non-specific sequences/chimaera removal using BLASTn abundance estimation as reference. We test HCK, Kraken, Qiime with ITSdb, Qiime with UNITE (Abarenkov et al., 2010a) and ITSdb database. DADA2 is tested only with the native UNITE database. The performance of each method is determined by its ability to assign the suitable taxa to the right sequence and to be able to assign the maximum of good sequences using sensitivity (recall), the positive predictive value (precision), and the f-score metric calculation (Figure 2). We determined True positive (TP) as following: For x_*i*_, the abundance estimated by the BLAST (reference) and *y_*i*_*, the abundance estimated by the tested methods for given sample *i*, we determined true positives by *TP_*i*_ = min(x_*i*_,y_*i*_)*. The overestimated abundance classified by the tested method is considered false positive, and the underestimation differences are included in the false negatives. The false negatives (FN) are determined by the sum of counts of amplicon only detected by BLAST but are not correctly assigned by the assessed method. For Tr_*i*_, the total abundance estimated by BLAST for given sample *i*, *FN_*i*_ = Tr_*i*_ − TP_*i*_*. The false positive (FP) corresponds to the sum of counts of amplicons wrongly assigned by the tested method but not detected by BLAST or not included in the initial mock community composition. For *Tm*_*i*_, the total number of amplicons classified for given taxa by the tested method, *FP_*i*_ = Tm_*i*_ – TP_*i*_*. We determined the precision (P_*i*_) and the recall (R_*i*_) and calculated the F- score using the following formula. *P_*i*_ = TP_*i*_/(TP_*i*_+FP_*i*_), R_*i*_ = TP_*i*_/(TP_*i*_+FN_*i*_)*, F-score_*i*_ = 2^∗^P_i^*^_R_*i*_/(P_*i*_ +R_*i*_) (Gardner et al., 2019). We also calculated alpha diversity using Shannon and chao1 indices to assess the association of chimaera removal methods and taxonomy classification in downstream diversity analysis. We compared it to the diversity of BLAST abundance estimation. We estimate the difference between the alpha diversity of the assessed methods and that of the BLASTn estimation. The lower the difference, the best is the method. All scripts and command lines are details in Supplementary Material: scripts_and_command_lines.

## Results

### Fungi ITS Amplicons Length: A Vast Diversity Among Species

**FIGURE 3:**
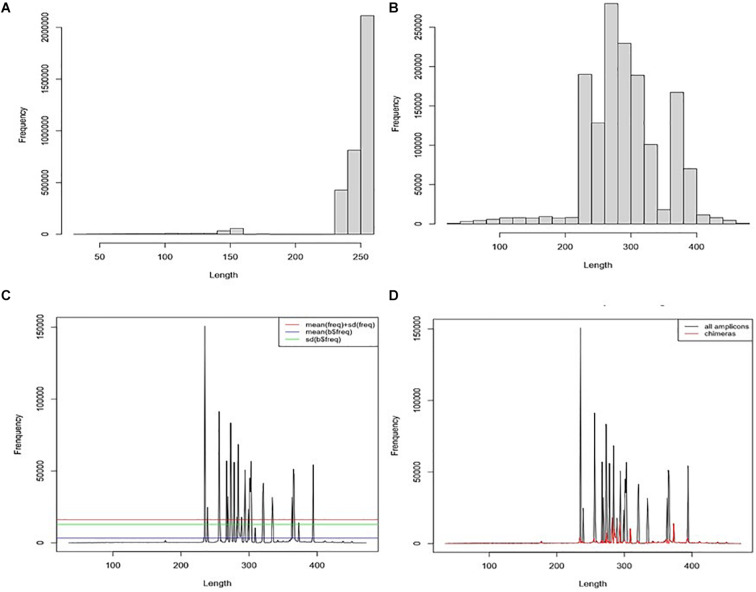
Chimaera detection flow using *ABAA*: **(A)** distribution of reads from all datasets; The average read length is 200.9 bp (*SD* = 65.6, a median of 250), the maximum reads size is 251 bp, and the minimum is 35 bp. **(B)** Distribution of the frequency of contigs length (assembly of forward and reverse reads), the average size is 233.3 bp (*SD* = 94.45) with a maximum of 472 bp and a minimum of 35 bp. The predominant amplicons size is 251 bps. **(C)** Distribution of contigs length-frequency by length: determination of non-specific amplicons filtering cut-off: cut-off was tested for means (blue line), standard deviation (green line), and mean + standard deviation (red line). The standard deviation was kept for better performance. **(D)** Distribution of sequences by the length in all datasets. Blackline represents the distribution of all sequences). Moreover, the red line represents the distribution of filtered sequences with ABAA (standard deviation).

All 11 samples from the three projects were combined into one single dataset during the pre-processing treatment. The average read length is 200.9 bp (*SD* = 65.6), the maximum read size is 251 bp, and the minimum is 35 bp (Figure 3A). Fragments with a read length below 150 bp have fewer duplicated percentages than those between 240 and 250. After joining the paired reads, the average size is 233.3 bp (*SD* = 94.45) with a maximum of 472 bp and a minimum of 35 bp. The predominant amplicons size is 251 bps. We observed low-frequency fragments below 250 bp and above 400 bp (Figure 3B).

### Taxonomic Assignment Using BLAST: Mock Communities Real Abundance Estimation as a Reference

We conducted a BLASTn search against the NCBI NT database to re-estimate the absolute abundance of the expected genus. We observed discrepancies between theoretical data and BLAST results. Even though samples SRR5439721 and SRR5439722 were from the same mock preparation, *Aspergillus* amplicons could not be detected in SRR5439721, and *Cryptococcus* was undermined in sample SRR5439721. *Malassezia* was also undermined in SRR6702280, SRR6702281, SRR6702283. The details of the abundance table are shown in Table 1.

### Benchmark and Comparative Analysis: Performance of HCK and ABAA

#### ABAA: Amplicons Filtering

For our analysis, amplicons length below 250 bp and above 400 bp have shown low frequency compared to those comprised between 251 and 400 bp (Figure 3D). Each peak in Figure 3C is composed of amplicons of a similar size. The enlargement of the base of the curve may correspond to the variation of the amplicon’s size. The frequency of these amplicons indicates that they could also be derived from non-specific amplification. Here we hypothesise this amplicon to be a chimaera and attempt to filter them out. The minimum sequence length detected by ABAA is 35 bp, with an average of 308 bp, higher than the overall average length (233 bp) and a maximum of 472 bp. It indicates that most chimaeras formed in this dataset may result from bimera and or multimera forming than incomplete amplification. Filtered amplicons by ABAA include amplicons below 250 bp, above 400 bp, and low amplification between 250 and 400 bp (Figure 3D). In total ABAA has detected 252,567 sequences accounting for 10.86% of overall sequences as non-specific amplicons while 528,544 (22.72%) with uchime_ref and 1,165,031 (50.08%) by DADA2 and 32 (0.0013%) detected by uchime_denovo. *isBimeraDenovo() in* DADA2 has filtered out up to 75.43% of sequences in sample SRR5439722. However, 24.76% were detected with UCHIME_REF, and 17.02% by ABAA on the other hand. Also, 51.74% were detected in sample SRR5439721, while 24.67% detected with UCHIME_REF and 17.23% by ABAA and UCHIME_REF seem to be more consistent than *isBimeraDenovo()* in DADA2 as samples SRR5439721 and SRR5439722 were from the same mock preparation (Table 2).

**TABLE 2:** Level of detection of chimaera removal methods.

Samples	Total sequences	Chimaera ABaa	Chimaera free Abaa*	Chimaera uchime_ref	Chimaera free uchime_ref*	Chimaera uchime_denovo	Chimaera free uchime_denovo*	Chimaera dada2	Chimaera free dada2*
SRR5439721	426,354	73,482 (17.23%)	352,872	105,167 (24.67%)	321,187	03 (00007%)	426,351	220,607 (51.74%)	205,747
SRR5439722	397,268	68,091 (17.02%)	329,177	98,367 (24.76%)	298,901	00 (0%)	397,268	299,665 (75.43%)	97,603
SRR6702280	174,185	19,187 (11.02%)	154,998	61,937 (35.56%)	112,248	11 (0.0063%)	174,174	71,129 (40.84%)	103,056
SRR6702281	148,397	17,980 (12.12%)	130,417	55,059 (37.10)	93,338	09 (0%)	148,388	63,247 (42.62%)	85,150
SRR6702283	246,368	31,616 (12.83%)	214,752	90,865 (36.88%)	155,503	08 (0.0032%)	246,360	93,543 (37.97%)	152,825
SRR8473974	865,248	40,730 (4.71%)	824,518	112,068 (12.95%)	753,180	01 (00001%)	865,247	388,352 (44.88%)	476,896
SRR8473977	17,181	379 (2.21%)	16,802	1,566 (9.11%)	15,615	00 (0%)	17,181	7,044 (41.00%)	10,137
SRR8473978	15,694	121 (0.77%)	15,573	1,363 (8.68%)	14,331	00 (0%)	15,694	6,510 (41.48%)	9,184
SRR8473979	18,394	295 (1.60%)	18,099	1,336 (7.26%)	17,058	00 (0%)	18,394	7,421 (40.34%)	10,973
SRR8473980	12,730	676 (5.31%)	12,054	387 (3.04%)	12,343	00 (0%)	12,730	4,898 (38.48%)	7,832
SRR8473984	4,420	10 (0.23%)	4,410	429 (9.71%)	3,991	00 (0%)	4,420	2,615 (59.16%)	1,805
Total	2,326,239	252,567 (10.86%)	2,073,672	528,544 (22.72%)	1,797,695	32 (0.0013%)	2,326,207	1,165,031 (50.08%)	1,161,208

*Sequence filtered with the corresponding method and cleaned.

#### HCK-ABAA: Taxonomic Assignment Performances

The second step of the HCK pipeline handles the chimaeras-free sequences. Samples sequences pre-processed and filtered by ABAA in the first step are then combined into a single fasta for the clustering process. With our datasets, we cluster a total of 2,326,239 amplicons with HCK using multiple-step iterated runs of cd-hit-est to perform hierarchical clustering. The first iteration performed with 99% sequence similarity generates 88,428 clusters, the second iteration with 98% creates 32,200 clusters (3/8 of the initial clusters), and the final iteration at 97% produces 18,831 clusters. The final iteration reduces the total clusters by 1/5 of the initial clusters, a crucial benefit of the hierarchical clustering that will be detailed in the discussion. All clusters generated by different iterations are merged into 18,770 non-redundant clusters, including 14,431 singletons, for which 2,545 have fragments size ≤ 149 bp and 11,886 with sequence size ≥150 bp (150 bp, widely considered as the minimum standard of ITS length). The singletons are removed from further analysis based on the assumption that a unique sequence might derive from sequencing errors or non-specific amplification. As a result, only 4,339 clusters are composed of biologically valid amplicons corresponding to 4,339 representative sequences. The performance of HCK with and without ABAA is assessed using the precision (positive predictive value), the recall (sensitivity), and the F-score based on the true-positive, false-positive, and false-negative rates as described in material and method. This performance is compared to other pipelines, e.g., QIIME version 1 with both databases ITSdb and UNITE, Kraken version1 with ITSdb, and DADA2 with UNITE database. All classification methods are tested with and without the chimaera removal step. The analysis shows that HCK without non-specific amplicons removal is slightly better than Kraken (precision: 0.685 and 0.682, recall: 0.986 and 0.983 and F-score: 0.80 and 0.79, respectively) and HCK decreases by 13.22% the false-positive detection and by 45.36% of false negatives compared to Kraken. The chimaera removal step with UCHIME_REF reduces the false positives by 32.05% and the false-negative by 10.44% compared to raw sequence processing. However, adding a step of chimaera filtering affects the sensitivity (recall), regardless of the method (Table 3).

**TABLE 3:** Precision, recall, accuracy, and F-score performance of HCK and ABAA version other tested combination of chimaera detection and taxonomy assignment methods.

Taxa. assign.	Chimaera remov.	Database	Total sequences	Unclassified sequences	True positive	False positive	False negative	Precision	Recall	F-score
hck	ABaa	ITSdb	2,073,672	378046	1,528,646	166,980	27,314	**0**.**834998**	0.97222	**0.89594**
hck	Uchime_ref	ITSdb	1,797,695	231779	1,306,938	258,978	249,022	0.803725	0.84344	0.81431
hck	Uchime_denovo	ITSdb	2,326,207	102360	1,547,646	676,201	8,314	0.670532	0.98645	0.79213
hck	–	ITSdb	2,326,239	220025	1,547,649	558,565	8,311	0.685258	**0.98645**	0.8013
kraken	ABaa	ITSdb	2,073,672	73480	1,497,681	502,511	58,279	0.713001	0.9651	0.81431
kraken	Uchime_ref	ITSdb	1797695	138673	1,277,882	381,140	278,078	0.725327	0.83483	0.77351
kraken	Uchime_denovo	ITSdb	2,326,207	141772	1,540,747	643,688	15,213	0.682455	0.98295	0.7977
kraken	–	ITSdb	2,326,239	141804	1,540,747	643,688	15,213	0.682455	0.98295	0.7977
qiime	ABaa	ITSdb	2,073,672	407960	1,369,677	296,035	186,283	0.782386	0.83022	0.78878
qiime	Uchime_ref	ITSdb	1,797,695	389342	1,175,806	232,547	380,154	0.765336	0.72615	0.71648
qiime	Uchime_denovo	ITSdb	2,326,207	515687	1,391,958	418,562	164,002	0.734174	0.83587	0.76968
qiime	–	ITSdb	2,326,239	525891	1,391,597	408,751	164,363	0.735705	0.83605	0.77068
qiime	ABaa	Unite	2,073,672	455026	1,208,721	409,925	347,239	0.529577	0.76841	0.62466
qiime	Uchime_ref	Unite	1,797,695	43024	1,050,365	704,306	505,595	0.510642	0.64894	0.56654
qiime	Uchime_denovo	Unite	2,326,207	347217	1,310,235	668,755	245,725	0.527798	0.79978	0.63181
qiime	–	Unite	2,326,239	52075	1,307,126	967,038	248,834	0.539557	0.79841	0.6396
Dada2		Unite	1,161,208	0	750,588	410,620	805,372	0.717852	0.5755	0.62471

*These values were highlighted in bold to show they are the top values.*

**FIGURE 4:**
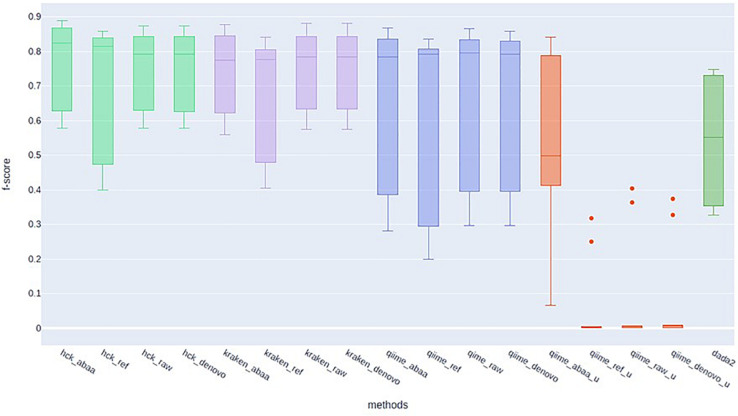
F-score performance of HCK (light green), Kraken (mauve), Qiime with ITSdb (blue), Qiime with Unite database (red), and DADA2 (green).

HCK yields better classification performance when ABAA is added upstream (precision 0.83 and the second best is HCK/UCHIME_REF with 0.8), and consequently, the F-score is also improved (0.89, Figure 4). Besides, the association of HCK and ABAA reduces the proportion of false-positive by 35.52% compared to HCK with UCHIME_REF and 97.01% without non-specific sequences removal. QIIME used with UCHIME_REF, and ITSdb performs better (F-score = 0.716) than similar approach with UNITE database (f-score = 0.648). DADA2 was also tested with its filtering method includes in the pipeline. The true-positive sequence classified was shallow compared to others, and this might be due to the high number of chimaeras sequences filtered (50.08%). Its F-score performance is 0.62, with 411 775 false-positive and 805 372 false negatives. The Kraken based classification implemented with chimaera methods yields comparable sensitivity results (recall) to that of HCK, but the higher number of false-positive impacts the precision and the overall performance (F-score: 0.79) (Table 3).

#### Diversity Metrics Analysis

**FIGURE 5:**
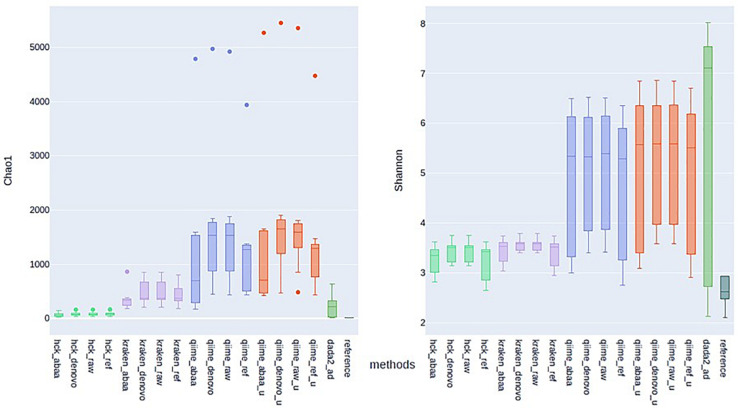
Estimation of Alpha diversity metric with Chao1 (left) and Shannon (right) indexes calculated with the estimated abundance of different methods and association with various chimaera removal methods

One of the most significant endpoints of ITS sequencing is the comparison of alpha diversity; thus, we compare the alpha diversity of all tested classification methods to that of BLASTn using Chao1 and Shannon indexes, assuming that diversity with BLAST search is closer to reality. With the Chao1 index, HCK diversity is closed to BLASTn estimation compared to Kraken, QIIME, and DADA2 estimation. With chao1, HCK in association with ABAA held the lowest difference with BLASTn (54.02), followed by HCK with UCHIME_DENOVO. With the Shannon index, HCK, used with UCHIME_REF, held the best rank(0.76), followed by HCK with ABAA (Supplementary Table 1). The data show that the BLASTn search estimates the chao1 index between 8 and 14 for all the samples. HCK with ABAA chaos1estimates is between 20 and 80, and kraken with and without chimaera removal’s estimation between 176 and 856. DADA2’s chao1 estimation is also close to the BLASTn search’s; however, there is an overestimation for some samples (15−650, Figure 5). The average Shannon index diversity of BLASTn search is 2 for all samples. It varies between 2 and 3 with HCK and ABAA, 2−7 for DADA2, 3 for Kraken with or without chimaera removal, and up to 6 for QIIME, depending on the database and the chimaera removal method (Figure 5).

#### Computing Specification and Speed

ABAA and HCK computing resources were tested and timed on ubuntu-based system 20.04, WSL2; Processor: Intel^®^ Core^TM^ i7-8650U CPU @ 1.90GHz 2.11 GHz; RAM 16.0 GB; System type 64-bit Operating System, x64-based processor. The running speed for each method tested is listed in Supplementary Table 2. ABaa filtered 2,326,239 sequences (623 MiB) in less than 2 min (1 min 18 s), while this requires almost 50 min for UCHIME_REF. *isBimeraDenovo()* function of DADA2 could not be tested separately as this step depends on many other steps. HCK and Qiime v1 process 23,700 sequences per min on average, while DADA2 processes 65,300 sequences per min. This speed does not include sequence truncation as it is not required for ITS processing.

## Discussion

In this work, we present full fungi ITS based classification workflow using two newly developed tools, ABAA and HCK, to filter non-specific amplicons and taxonomically classify them. We compare the performance of ABAA to UCHIME and *isBimeraDenovo() in* DADA2 and the performance of HCK to that of Kraken, QIIME, and DADA2 using publicly available mock community Illumina sequencing datasets. The analysis revealed that HCK-ABAA yields the best performance. The F-score is systematically improved when ABAA is used to filter amplicons regardless of the classifier. This work also shows the impact of filtering methods on the ecological diversity metrics and how they can dramatically change the estimation of a sample’s diversity.

The efficiency of sequence amplification and the quality of sequencing reads are critical and determinants for the outcome of the metabarcoding analysis and especially for fungi ITS locus (Schloss et al., 2011). Non-specific amplicons present a serious threat to the classification and taxa abundance estimation. The size and the number of ITS loci are highly variable, unlike the 16S rRNA gene in bacteria and sufficiently polymorphic to delineate fungi at the genus and or species level (Tang et al., 2015; Khodadadi et al., 2017). This variation can be biological or can derive from high rates of insertions and deletions in the evolution of this less conserved genetic region. It can also derive from non-specific amplification. ABAA has this advantage of considering the real distribution of amplicons size from real datasets. It does not need database maintenance and only requires minimum computing resources. It filters sequences based on the distribution of their size-frequency and mainly targets amplicons with low length-frequency. The performance of the majority of chimaera filtering methods are usually assessed on simulated chimaera sequences (Nilsson et al., 2010; Harris et al., 2012), but when applied to the real dataset, it is challenging to determine whether sequences that have been filtered are real chimaeras. The fragment size dissimilarity also creates bias during conventional clustering. Consequently, this affects OTUs picking and abundance estimation, including overestimating or underestimating community abundance (De Filippis et al., 2017). Except for Kraken, the majority of metabarcoding methods include a clustering process. Clustering consists of reducing the amplicons similarity redundancy of data diversity. The most commonly used in amplicon metabarcoding analysis are uclust in the usearch algorithm (Edgar, 2010), vsearch (Rognes et al., 2016), and CD-HIT (Fu et al., 2012). usearch and vsearch can cluster nucleotide sequences based on their similarity, length, and abundance, assuming that the same species’ amplicons will probably be identical in size with a minimal coverage dissimilarity. As a result, with fungi ITS, clustering may create multiple OTUs from the same species amplicons and increase the alpha and the beta diversities.

CD-HIT implements a more realistic clustering approach, hierarchical clustering, which consists of a multiple-step, iterated runs with a neighbour-joining approach and generates a hierarchical structure. In HCK, with the datasets that we analyse, the second iteration with 98% identity reduces the first number of clusters by 3/8 and the final iteration with 97% identity by 1/5. In addition to filtering out the singleton, HCK drastically reduces the number of false-positive and normalises the diversity abundance. It is essential to highlight that databases also play an important role in the performance of the classifier. Qiime version 1 performs better with the ITSdb database than its native database UNITE, regardless of the filtering method. The inappropriate estimation of the abundance (overestimation, underestimation of population or sequence wrongly classified) can also influence metrics of diversity. The high diversity found with the UNITE database might be due to the higher number of incorrect classification sequences in the UNITE database.

## Conclusion

The classification of fungi using ITS marker is very challenging. It is owed to the high diversity of the kingdom. Moreover, targeting an intergenic section as ITS1 leads to diversified amplicon sizes and sequences that are not taken into account with the classical approaches developed for 16S analysis. Combining HCK and ABAA increases the number of true-positive and decreases the proportion of false-positive, as shown with the datasets we have evaluated. Consequently, HCK maintained the alpha diversity metric with the Chao1 index close to that of the BLASTn, compared to QIIME, Kraken, and DADA2. As demonstrated in this analysis, the use of HCK in association with ABAA allows a more realistic estimation of fungal diversity. So far, it is the best option to perform fungi ITS1 metabarcoding analysis on clinical and non-clinical samples.

## Data Availability Statement

Sequences analysed in this project are available under accession numbers SRR8473974, SRR8473977, SRR8473978, SRR8473979, SRR8473980, SRR8473984, SRP132544, SRR6702280, SRR6702281, SRR6702283, SRR5439721, and SRR5439722 in NCBI. The original contributions presented in the study are included in the Material and method section, further inquiries can be directed to the corresponding authors.

## Author Contributions

AD and OP supervised the study. KM analysed interpreted data and wrote the manuscript. CB, AM, AO, and AK made critical revisions. All authors read and approved the final manuscript.

## Conflict of Interest

AO, AK and OP were employed by the company L’Oreal. The remaining authors declare that the research was conducted in the absence of any commercial or financial relationships that could be construed as a potential conflict of interest.

## Footnotes

1 ftp.ncbi.nlm.nih.gov/refseq/TargetedLoci/Fungi/

2 https://github.com/bakerccm/entrez_qiime

## References

[B1] Abarenkov K. Henrik Nilsson R. Larsson K.-H. Alexander I. J. Eberhardt U. Erland S. (2010a). The UNITE database for molecular identification of fungi - recent updates and future perspectives. *New Phytol.* 186 281–285. 10.1111/j.1469-8137.2009.03160.x 20409185

[B2] Abarenkov K. Tedersoo L. Nilsson R. H. Vellak K. Saar I. Veldre V. (2010b). Plutof-a web-based workbench for ecological and taxonomic research, with an online implementation for fungal its sequences. *Evol. Bioinform.* 6 189–196. 10.4137/EBO.S6271

[B3] Bazzicalupo A. L. Bálint M. Schmitt I. (2013). Comparison of ITS1 and ITS2 rDNA in 454 sequencing of hyperdiverse fungal communities. *Fungal Ecol.* 6 102–109. 10.1016/j.funeco.2012.09.003

[B4] Bellemain E. Carlsen T. Brochmann C. Coissac E. Taberlet P. Kauserud H. (2010). ITS as an environmental DNA barcode for fungi: an in silico approach reveals potential PCR biases. *BMC Microbiol.* 10:189. 10.1186/1471-2180-10-189 20618939 PMC2909996

[B5] Bjørnsgaard Aas A. Davey M. L. Kauserud H. (2017). ITS all right mama: investigating the formation of chimeric sequences in the ITS2 region by DNA metabarcoding analyses of fungal mock communities of different complexities. *Mol. Ecol. Resour.* 17 730–741. 10.1111/1755-0998.12622 27775220

[B6] Blaalid R. Kumar S. Nilsson R. H. Abarenkov K. Kirk P. M. Kauserud H. (2013). ITS1 versus ITS2 as DNA metabarcodes for fungi. *Mol. Ecol. Resour.* 13 218–224. 10.1111/1755-0998.12065 23350562

[B7] Bolger A. M. Lohse M. Usadel B. (2014). Trimmomatic: a flexible trimmer for Illumina sequence data. *Bioinformatics* 30 2114–2120. 10.1093/bioinformatics/btu170 24695404 PMC4103590

[B8] Bolyen E. Rideout J. R. Dillon M. R. Bokulich N. A. Abnet C. Al-Ghalith G. A. (2018). QIIME 2: reproducible, interactive, scalable, and extensible microbiome data science. *Nat Biotechnol.* 37 852–857. 10.7287/peerj.preprints.27295v1 PMC7015180 31341288

[B9] Callahan B. J. McMurdie P. J. Holmes S. P. (2017). Exact sequence variants should replace operational taxonomic units in marker-gene data analysis. *ISME J.* 11 2639–2643. 10.1038/ismej.2017.119 28731476 PMC5702726

[B10] Callahan B. J. McMurdie P. J. Rosen M. J. Han A. W. Johnson A. J. A. Holmes S. P. (2016). DADA2: high-resolution sample inference from Illumina amplicon data. *Nat. Methods* 13 581–583. 10.1038/nmeth.3869 27214047 PMC4927377

[B11] Caporaso J. G. Kuczynski J. Stombaugh J. Bittinger K. Bushman F. D. Costello E. K. (2010). QIIME allows analysis of high-throughput community sequencing data. *Nat. Methods* 7 335–336. 10.1038/nmeth.f.303 20383131 PMC3156573

[B12] De Filippis F. Laiola M. Blaiotta G. Ercolini D. (2017). Different amplicon targets for sequencing-based studies of fungal diversity. *Appl. Environ. Microbiol.* 83:e00905-17. 10.1128/AEM.00905-17 28625991 PMC5561290

[B13] Edgar R. (2016). UCHIME2: improved chimera prediction for amplicon sequencing. *bioRxiv* [Preprint] 10.1101/074252

[B14] Edgar R. C. (2010). Search and clustering orders of magnitude faster than BLAST. *Bioinformatics* 26 2460–2461. 10.1093/bioinformatics/btq461 20709691

[B15] Fosso B. Santamaria M. Marzano M. Alonso-Alemany D. Valiente G. Donvito G. (2015). BioMaS: a modular pipeline for bioinformatic analysis of metagenomic AmpliconS. *BMC Bioinform.* 16:203. 10.1186/s12859-015-0595-z 26130132 PMC4486701

[B16] Fu L. Niu B. Zhu Z. Wu S. Li W. (2012). CD-HIT : accelerated for clustering the next-generation sequencing data. *Bioinformatics* 28 3150–3152. 10.1093/bioinformatics/bts565 23060610 PMC3516142

[B17] Fujita S. I. Senda Y. Nakaguchi S. Hashimoto T. (2001). Multiplex PCR using internal transcribed spacer 1 and 2 regions for rapid detection and identification of yeast strains. *J. Clin. Microbiol.* 39 3617–3622. 10.1128/JCM.39.10.3617-3622.2001 11574582 PMC88398

[B18] Gardner P. P. Watson R. J. Morgan X. C. Draper J. L. Finn R. D. Morales S. E. (2019). Identifying accurate metagenome and amplicon software via a meta-analysis of sequence to taxonomy benchmarking studies. *PeerJ.* 7:e6160. 10.7717/peerj.6160 30631651 PMC6322486

[B19] Gweon H. S. Oliver A. Taylor J. Booth T. Gibbs M. Read D. S. (2015). PIPITS: an automated pipeline for analyses of fungal internal transcribed spacer sequences from the Illumina sequencing platform. *Methods Ecol. Evol.* 6 973–980. 10.1111/2041-210X.12399 27570615 PMC4981123

[B20] Harris S. R. Clarke I. N. Seth-Smith H. M. B. Solomon A. W. Cutcliffe L. T. Marsh P. (2012). Whole-genome analysis of diverse Chlamydia trachomatis strains identifies phylogenetic relationships masked by current clinical typing. *Nat. Genet.* 44 413–419, S1. 10.1038/ng.2214 22406642 PMC3378690

[B21] Hoggard M. Vesty A. Wong G. Montgomery J. M. Fourie C. Douglas R. G. (2018). Characterising the human mycobiota: a comparison of small subunit rRNA, ITS1, ITS2, and large subunit rRNA genomic targets. *Front. Microbiol.* 9:2208. 10.3389/fmicb.2018.02208 30283425 PMC6157398

[B22] Khodadadi H. Karimi L. Jalalizand N. Adin H. Mirhendi H. (2017). Utilisation of size polymorphism in ITS1 and ITS2 regions for identification of pathogenic yeast species. *J. Med. Microbiol.* 66 126–133. 10.1099/jmm.0.000426 28260588

[B23] Kim B.-R. Shin J. Guevarra R. B. Lee J. H. Kim D. W. Seol K.-H. (2017). Deciphering diversity indices for a better understanding of microbial communities. *J. Microbiol. Biotechnol* 27 2089–2093. 10.4014/jmb.1709.09027 29032640

[B24] Kõljalg U. Nilsson R. H. Abarenkov K. Tedersoo L. Taylor A. F. S. Bahram M. (2013). Towards a unified paradigm for sequence-based identification of fungi. *Mol. Ecol.* 22 5271–5277. 10.1111/mec.12481 24112409

[B25] Kumar S. Carlsen T. Mevik B. H. Enger P. Blaalid R. Shalchian-Tabrizi K. (2011). CLOTU: an online pipeline for processing and clustering of 454 amplicon reads into OTUs followed by taxonomic annotation. *BMC Bioinform.* 12:182. 10.1186/1471-2105-12-182 21599929 PMC3120705

[B26] Lahr D. J. G. Katz L. A. (2009). Reducing the impact of PCR-mediated recombination in molecular evolution and environmental studies using a new-generation high-fidelity DNA polymerase. *Biotechniques* 47 857–866. 10.2144/000113219 19852769

[B27] Martin K. J. Rygiewicz P. T. (2005). Fungal-specific PCR primers developed for analysis of the ITS region of environmental DNA extracts. *BMC Microbiol.* 5:28. 10.1186/1471-2180-5-28 15904497 PMC1156903

[B28] Masella A. P. Bartram A. K. Truszkowski J. M. Brown D. G. Neufeld J. D. (2012). PANDAseq: paired-end assembler for illumina sequences. *BMC Bioinformatics* 13:31. 10.1186/1471-2105-13-31 22333067 PMC3471323

[B29] McTaggart L. R. Copeland J. K. Surendra A. Wang P. W. Husain S. Coburn B. (2019). Mycobiome sequencing and analysis applied to fungal community profiling of the lower respiratory tract during fungal pathogenesis. *Front. Microbiol.* 10:512. 10.3389/fmicb.2019.00512 30930884 PMC6428700

[B30] Mysara M. Vandamme P. Props R. Kerckhof F. M. Leys N. Boon N. (2017). Reconciliation between operational taxonomic units and species boundaries. *FEMS Microbiol. Ecol.* 93:fix029. 10.1093/femsec/fix029 28334218 PMC5812548

[B31] Nilsson R. H. Abarenkov K. Veldre V. Nylinder S. de Wit P. Brosché S. (2010). An open source chimera checker for the fungal ITS region. *Mol. Ecol. Resour.* 10 1076–1081. 10.1111/j.1755-0998.2010.02850.x 21565119

[B32] Nilsson R. H. Ryberg M. Abarenkov K. Sjökvist E. Sjökvist S. Kristiansson E. (2008). The ITS region as a target for characterisation of fungal communities using emerging sequencing technologies. *FEMS Microbiol. Lett.* 296 97–101. 10.1111/j.1574-6968.2009.01618.x 19459974

[B33] Ninet B. Jan I. Bontems O. Léchenne B. Jousson O. Panizzon R. (2003). Identification of dermatophyte species by 28S ribosomal DNA sequencing with a commercial kit. *J. Clin. Microbiol.* 41 826–830. 10.1128/JCM.41.2.826-830.2003 12574293 PMC149666

[B34] Rognes T. Flouri T. Nichols B. Quince C. Mahé F. (2016). VSEARCH: a versatile open source tool for metagenomics. *PeerJ* 4:e2584. 10.7717/peerj.2584 27781170 PMC5075697

[B35] Schloss P. D. Gevers D. Westcott S. L. (2011). Reducing the {Effects} of {PCR} {Amplification} and {Sequencing} {Artifacts} on 16S {rRNA}-{Based} {Studies}. *PLoS One* 6:e27310. 10.1371/journal.pone.0027310 22194782 PMC3237409

[B36] Schloss P. D. Westcott S. L. Ryabin T. Hall J. R. Hartmann M. Hollister E. B. (2009). Introducing mothur: open-source, platform-independent, community-supported software for describing and comparing microbial communities. *Appl. Environ. Microbiol.* 75 7537–7541. 10.1128/AEM.01541-09 19801464 PMC2786419

[B37] Tang J. Iliev I. D. Brown J. Underhill D. M. Funari V. A. (2015). Mycobiome: approaches to analysis of intestinal fungi. *J. Immunol. Methods* 421 112–121. 10.1016/j.jim.2015.04.004 25891793 PMC4451377

[B38] Wang X. C. Liu C. Huang L. Bengtsson-Palme J. Chen H. Zhang J. H. (2015). ITS1: a DNA barcode better than ITS2 in eukaryotes? *Mol. Ecol. Resour.* 15 573–586. 10.1111/1755-0998.12325 25187125

[B39] White J. R. Maddox C. White O. Angiuoli S. V. Fricke W. F. (2013). CloVR-ITS: automated internal transcribed spacer amplicon sequence analysis pipeline for the characterisation of fungal microbiota. *Microbiome* 1:6. 10.1186/2049-2618-1-6 24451270 PMC3869194

[B40] Wood D. E. Salzberg S. L. (2014). Kraken: ultrafast metagenomic sequence classification using exact alignments. *Genome Biol.* 15:R45 10.1186/gb-2014-15-3-r46 24580807 PMC4053813

[B41] Wu S. Xiong J. Yu Y. (2015). Taxonomic resolutions based on 18S rRNA genes: a case study of subclass copepoda. *PLoS One* 10:e0131498. 10.1371/journal.pone.0131498 26107258 PMC4479608

[B42] Zajec N. Stres B. Avguštin G. (2012). Distinct approaches for the detection and removal of chimeric 16S rRNA sequences can significantly affect the outcome of between-site comparisons. *Aquat. Microb. Ecol.* 66 13–21. 10.3354/ame01510

