# Supplementary material for: HCK and ABAA: A Newly Designed Pipeline to Improve Fungi Metabarcoding Analysis
Source: Front Microbiol. 2021 May 5;12:640693. doi: 10.3389/fmicb.2021.640693 (PMC8134036; doi:10.3389/fmicb.2021.640693)
Supplement: Supplementary file 1 [file Data_Sheet_1.docx]

The size of fungal ITS1 sequences is highly variable and can differ from one species to another. The sequence length dissimilarity as well as chimaera amplicons generated during Polymerase Chain Reaction (PCR) create bias during similarity clustering and consequently affect community abundance estimation in the downstream analysis. This script manual instructs the pre-processing and processing steps of a comparative analysis of a newly designed pipeline, The Hierarchical Clustering with Kraken (HCK), implemented with Abundance-Base Alternative Approach (ABaa) to filter chimeric sequences and classify ITS1 amplicons in fungi metabarcoding datasets versus existing Qiime version1, DADA2 and Kraken version.

The database used for reference filtering was downloaded from the following link (<https://unite.ut.ee/sh_files/sh_qiime_release_31.01.2016.zip>).

The NCBI NT, and taxdb database downloaded locally from the following link (<https://ftp.ncbi.nlm.nih.gov/blast/db/>) released on the 2020-09-14.

UNITE database (UNITE Community 2019) downloaded from <http://unite.ut.ee/repository.php> for QIIME and DADA2. In this study, we used the version 8.2 released on the 2018-11-18 containing 9407 reference sequences and 26260 representative sequences.

1. Illumina reads sequences quality check

# install fastqc and multiqc locally

*mkdir -p QC_bf*

*fastqc -o QC_bf -f fastq --extract raw_data/*.fastq*

*multiqc -o merged_QC -f -v QC_bf*

1. Illumina raw sequence trimming

module load trimmomatic/0.36 or install locally trimmomatic (minimum version 0.35)

*mkdir $1*

*# $1=output_dir(trim),*

*$2=raw_data*

*for file in $(ls $2 | sed 's/R[12]\.fastq//')*

*do*

*trimmomatic PE -phred33 \*

*$2/${file}R1.fastq $2/${file}R2.fastq \*

*$1/${file}R1.paired.fastq $1/${file}R1.unpaired.fastq $1/${file}R2.paired.fastq $1/${file}R2.unpaired.fastq \*

*ILLUMINACLIP:$HOME/my_env/TruSeq3-PE.fa:2:30:10 LEADING:3 TRAILING:3 SLIDINGWINDOW:4:15 \*

*-threads 30 \*

*MINLEN:50*

*done*

1. Merging forward and reverse sequence reads

module load pandaseq/2.11 or install locally pandaseq (minimum version 2.10)

*mkdir -p $1 ## $1=output_dir, $2=trimmed_fastq.gz*

*for file in $(ls $2/*.paired.fastq | cut -f2 -d "/" | sed 's/\.R[12].paired\.fastq//');*

*do*

*pandaseq \*

*-f $2/${file}.R1.paired.fastq \*

*-r $2/${file}.R2.paired.fastq \*

*-N -l 50 -o 20 -B -t 0.9 -T 50 \*

*-A simple_bayesian -w $1/${file}.fasta;*

*done*

1. Combining all samples fasta files into one single fasta file (demultiplexing)

*add_label.py -i raw_fasta -o SEQ/raw_fasta*

1. Non-specific amplicons detection with Abaa

*abaa.py -i input_dir -o output_dir* ## input_dir should contain amplicons fasta files

1. Chimera filtering with UCHIME_REF and UCHIME_DENOVO

*vsearch --uchime_denovo SEQ/raw.fasta --threads 20 --nonchimeras SEQ/denovo.fasta & vsearch --uchime_ref SEQ/raw.fasta --threads 40 --db $silva_ref --nonchimeras SEQ/ref.fasta*

1. Blast search

*blastn -query SEQ/raw.fasta -out BLAST/All.tsv -task megablast -strand both -db $DB/nt -num_threads 10 -evalue 1e-10 -outfmt "6 qseqid qlen qstart qend sseqid slen sstart send pident evalue qcovs bitscore staxids sskingdoms sscinames" -max_target_seqs 100*

1. DADA2 adapted for fungi ITS amplicons

#preparing the working environment

*args = commandArgs(trailingOnly=TRUE)*

*library(dada2)*

*packageVersion("dada2")*

*library(ShortRead)*

*packageVersion("ShortRead")*

*library(Biostrings)*

*packageVersion("Biostrings")*

*library(phyloseq)*

*packageVersion("phyloseq")*

# load reads files

*path <- args[1]*

*#list.files(path)*

#match foreward and reverse files

*fnFs <- sort(list.files(path, pattern = "R1.fastq.gz", full.names = TRUE))*

*fnRs <- sort(list.files(path, pattern = "R2.fastq.gz", full.names = TRUE))*

# Forward and reverse fastq filenames have the format:

*cutFs <- sort(list.files(path, pattern = "R1.fastq.gz", full.names = TRUE))*

*cutRs <- sort(list.files(path, pattern = "R2.fastq.gz", full.names = TRUE))*

# Extract sample names, assuming filenames have format:

*get.sample.name <- function(fname) strsplit(basename(fname), "_")[[1]][1]*

*sample.names <- unname(sapply(cutFs, get.sample.name))*

*#head(sample.names)*

#triming

*filtFs <- file.path(path, "filtered", basename(cutFs))*

*filtRs <- file.path(path, "filtered", basename(cutRs))*

*out <- filterAndTrim(cutFs, filtFs, cutRs, filtRs, maxN = 0, maxEE = c(2, 2), truncQ = 2, minLen = 50, rm.phix = TRUE, compress = TRUE, multithread = TRUE) # on windows, set multithread = FALSE*

*errF <- learnErrors(filtFs, multithread = TRUE)*

*errR <- learnErrors(filtRs, multithread = TRUE)*

#dereplicate

*derepFs <- derepFastq(filtFs, verbose = TRUE)*

*derepRs <- derepFastq(filtRs, verbose = TRUE)*

# Name the derep-class objects by the sample names

*names(derepFs) <- sample.names*

*names(derepRs) <- sample.names*

#sample inference

*dadaFs <- dada(derepFs, err = errF, multithread = TRUE)*

*dadaRs <- dada(derepRs, err = errR, multithread = TRUE)*

#merging

*mergers <- mergePairs(dadaFs, derepFs, dadaRs, derepRs, verbose=TRUE)*

#sequence table

*seqtab <- makeSequenceTable(mergers)*

#remove chimeras

*seqtab.nochim <- removeBimeraDenovo(seqtab, method="consensus", multithread=TRUE, verbose=TRUE)*

#track reads

*getN <- function(x) sum(getUniques(x))*

*track <- cbind(out, sapply(dadaFs, getN), sapply(dadaRs, getN), sapply(mergers, getN), rowSums(seqtab.nochim))*

# If processing a single sample, remove the sapply calls: e.g. replace

*# sapply(dadaFs, getN) with getN(dadaFs)*

*colnames(track) <- c("input", "filtered", "denoisedF", "denoisedR", "merged", "nonchim")*

*rownames(track) <- sample.names*

*write.table(track, "track.tsv")*

#taxonomy

*unite.ref <- args[2]*

*taxa <- assignTaxonomy(seqtab.nochim, unite.ref, multithread = TRUE, tryRC = TRUE)*

*taxa.print <- taxa # Removing sequence rownames for display only*

*rownames(taxa.print) <- NULL*

*write.table(taxa, "taxa.tsv")*

*write.table(taxa.print, "taxa_print.tsv")*

#export otu table

*ps <- phyloseq(otu_table(seqtab.nochim, taxa_are_rows=FALSE), tax_table(taxa))*

*dna <- Biostrings::DNAStringSet(taxa_names(ps))*

*names(dna) <- taxa_names(ps)*

*ps <- merge_phyloseq(ps, dna)*

*taxa_names(ps) <- paste0("ASV", seq(ntaxa(ps)))*

*t = t(otu_table(ps))*

*write.table(t, "otu_table.tsv")*

*t2 = tax_table(ps)*

*write.table(t2, "taxa_table.tsv")*

## users can make a script out of these command lines and name it dada2.R, he will simply need to run thefollowing command

*Rsripts dada2.R arg1 (raw_data_dir) arg2 (Unite_db_dir)*

1. QIIME version 1 scripts

#unite_db

*for i in $(ls SEQ/ | sed 's/\.fasta//'); do pick_open_reference_otus.py -i SEQ/$i.fasta -r $unite_ref -o QIIME_$i -p $unite_setting -f -a -O 5 -v; summarize_taxa.py -i QIIME_$i/otu_table_mc2.biom -a -o QIIME_$i/tax -L 1,2,3,4,5,6,7; done*

#unite_setting

pick_otus:enable_rev_strand_match True

assign_taxonomy:reference_seqs_fp dir_to_Unite_db

assign_taxonomy:id_to_taxonomy_fp dir_to_Unite_taxa_file

#ITSdb

*for i in $(ls SEQ/ | sed 's/\.fasta//'); do pick_open_reference_otus.py -i SEQ/$i.fasta -r fungi_QIIME_DB.fasta.sorted -o QIIME_$i -a -O 2 -p fungi_setting.txt -f -v; summarize_taxa.py -i QIIME_$i/otu_table_mc2_w_tax.biom -a -o QIIME_$i/tax -L 1,2,3,4,5,6,7; done*

#itsdb_setting

*pick_otus:enable_rev_strand_match True*

*assign_taxonomy:reference_seqs_fp dir_to_ITSdb*

*assign_taxonomy:id_to_taxonomy_fp dir_ITSdb_taxa_file*

1. **Kraken analysis**

*mkdir -p $1* #create results directory

*in=$2* # fasta file

*db=$3* # db_dir

*kraken --db $db --preload --unclassified-out $1/unclassified.fna --classified-out $1/classified.fna $in > $1/metagenome.kraken*

#generate representative report

*kraken-report $1/metagenome.kraken --db $db > $1/metagenome.report*

*ls fasta |sed 's/\.fasta//g' > $1/list.txt* # fasta is the directory containing the list of samples fasta files, the aim is to generate the sample list name

#generate kraken profile for all study samples

*cd $1*

*mkdir -p sample*

*cat list.txt | while read line; do grep $line metagenome.kraken > sample/$line.kraken; echo "just finish $line"; done*

#generate kraken report for all samples

*mkdir -p report*

*mkdir -p abundance*

*for file in $(ls sample); do d="$(echo ${file} | sed 's/kraken/report/')"; kraken-report sample/${file} --db $db > report/$d; echo "just finish $d"; done*

#plot abundance

*Rscript filter2.R report abundance*

*echo "All done...!"*

# users can make a script out of theses command lines , name it kraken.sh and run the following command:

*bash kraken.sh $1(results_dir) $2 (input_dir) $3(db_dir)*

1. Alpha diversity

*alpha_diversity.py -i QIIME/otu_table_mc2.biom -m chao1,shannon -o adiv_chao1_shan.tsv*
