# Supplementary material for: HCK and ABAA: A Newly Designed Pipeline to Improve Fungi Metabarcoding Analysis
Source: Front Microbiol. 2021 May 5;12:640693. doi: 10.3389/fmicb.2021.640693 (PMC8134036; doi:10.3389/fmicb.2021.640693)
Supplement: Supplementary file 2 [file Table_1.docx]

Supplementary material

Table 1: The arithmetic difference between BLASTnn search and assessed methods alpha diversity (Chao1 and Shannon indexes)

|  | dada2 | Hck  abaa | Hck  denovo | Hck  raw | Hck  ref | Kraken  abaa | Kraken  denovo | Kraken  raw | Kraken  ref | Qiime  abaa_u | Qiime  denovo_u | Qiime  raw_u | Qiime  abaa | Qiime  denovo | Qiime  raw | qiime_ref |
| --- | --- | --- | --- | --- | --- | --- | --- | --- | --- | --- | --- | --- | --- | --- | --- | --- |
| Chao1 | 195.38 | 54.02 | 73.90 | 73.34 | 76.05 | 338.67 | 470.57 | 470.57 | 421.63 | 1287.31 | 1750.29 | 1735.72 | 1139.11 | 1553.51 | 1556.62 | 1185.97 |
| Shannon | 3.06 | 0.81 | 0.96 | 0.96 | 0.76 | 1.00 | 1.10 | 1.10 | 0.95 | 2.54 | 2.83 | 2.84 | 2.41 | 2.67 | 2.69 | 2.33 |

Table 2: ABAA and HCK running time and speed

| Methods | Running time | Average speed |
| --- | --- | --- |
|  |  |  |
| ABAA | 1 min 18s | 1789500 seqs /min |
| UCHIME_REF | 49 min 19 s | 47200 seqs/min |
|  |  |  |
| HCK | 85 min 14s | 27300 seqs/min |
| QIIME | 85 min 42 s | 27300 seqs/min |
| DADA2* | 35 min 39s | 65300 seqs/min |
| KRAKEN | 7 min 10 s | 290800 seqs/min |

*process includes trimming, filtering, denoising and chimaera filtering
